# Differential Impact of Medical Therapies for Acromegaly on Glucose Metabolism

**DOI:** 10.3390/ijms26020465

**Published:** 2025-01-08

**Authors:** Federico Gatto, Anna Arecco, Jessica Amarù, Marica Arvigo, Claudia Campana, Angelo Milioto, Daniela Esposito, Gudmundur Johannsson, Francesco Cocchiara, Davide Carlo Maggi, Diego Ferone, Alessandra Puddu

**Affiliations:** 1 Endocrinology Unit, Department of Internal Medicine and Medical Specialties, School of Medical and Pharmaceutical Sciences, University of Genova, 16132 Genova, Italy; 2 Endocrinology Unit, IRCCS Ospedale Policlinico San Martino, 16132 Genova, Italy; 3 Department of Internal Medicine and Clinical Nutrition, Institute of Medicine, Sahlgrenska Academy, University of Gothenburg, 40530 Gothenburg, Sweden; 4 Department of Endocrinology, Sahlgrenska University Hospital, 41345 Gothenburg, Sweden

**Keywords:** blood glucose, hyperglycaemia, diabetes mellitus, somatostatin receptor ligands, octreotide, lanreotide, pasireotide, growth hormone receptor antagonists, pegvisomant, dopamine agonists

## Abstract

Acromegaly is a rare endocrine disorder caused by excessive growth hormone (GH) production, due, in the vast majority of cases, to the presence of a GH-secreting pituitary tumour. The chronic elevation of GH and the resulting high circulating levels of insulin-like growth factor-1 (IGF-1) cause the characteristic tissue overgrowth and a number of associated comorbidities, including several metabolic changes, such as glucose intolerance and overt diabetes mellitus (DM). Elevated GH concentrations directly attenuate insulin signalling and stimulate lipolysis, decreasing glucose uptake in peripheral tissues, thus leading to the development of impaired glucose tolerance and DM. Acromegaly treatment aims to normalize plasma GH and IGF-1 levels using surgery, medical treatment, or radiotherapy. The effect of the different medical therapies on glucose homeostasis varies. This literature review explores the impact of the currently available pharmacological therapies for acromegaly (first- and second-generation somatostatin receptor ligands, a GH receptor antagonist, and dopamine agonists) on glucose homeostasis. We also discuss the underlying biological mechanisms through which they impact glucose metabolism.

## 1. Introduction

Acromegaly is a rare and severe endocrine disorder caused by excessive growth hormone (GH) production, mostly due to a somatotroph pituitary tumour [1]. Other (extremely) rare causes of acromegaly are ectopic GH excess, produced by tumours other than pituitary adenomas (e.g., lymphomas and pancreatic islet cell tumours), as well as growth hormone-releasing hormone (GHRH) excess, secondary to lesions of the central nervous system (e.g., hypothalamic hamartomas, choristomas, and ganglioneuromas), or neuroendocrine neoplasms (e.g., carcinoid tumours and small cell lung cancers) [1]. The chronic elevation of GH and the resulting overproduction/hypersecretion of insulin-like growth factor-1 (IGF-1) cause the characteristic tissue overgrowth resulting in typical facial features, enlarged hands and feet, as well as various comorbidities affecting different functions (e.g., cardiovascular, osteoarticular, neurological and respiratory) and excess mortality [2,3]. GH and IGF-1 excess also causes several metabolic changes, including glucose intolerance and overt diabetes mellitus (DM). Prediabetes, such as impaired fasting glucose (IFG) and impaired glucose intolerance (IGT), is more frequent in patients with acromegaly than the general population [4,5], and the prevalence of DM ranges between 19% and 56% of acromegaly patients [6]. High circulating GH concentrations suppress glucose oxidation and its use in the whole body while enhancing hepatic gluconeogenesis [7]. Moreover, GH promotes fat metabolism by enhancing lipolysis and fatty acid oxidation, potentiating hormone-sensitive lipase activity by β-adrenergic stimulation [7]. Compensatory insulin hypersecretion, in response to insulin resistance, may lead to pancreatic β-cell dysfunction and a decline in insulin secretion, thus leading to the development of prediabetes and DM. It is noteworthy that patients with acromegaly are characterized by a peculiar phenotype combining insulin resistance and increased lean body mass [8].

DM has a negative impact on overall mortality and cardiovascular mortality and morbidity in acromegaly [6]; therefore, it is important to prevent DM and personalize medical therapy for each patient. Acromegaly treatments aim to normalize plasma GH and IGF-1 concentrations, improving insulin resistance and reducing gluconeogenesis. Transsphenoidal surgery is the first line of therapy in most cases [9]. When surgery is not curative (about half of the cases), or if it is contraindicated or refused by the patient, medical therapy using somatostatin receptor ligands (SRLs) is recommended [9]. Radiotherapy is usually reserved for patients who have failed, are unfit for, or have declined surgical and/or medical therapy [9]. The effects on glucose homeostasis vary depending on therapy type: a successful surgery that lowers plasma GH and normalizes IGF-1 concentrations has a positive effect [10], while some medical therapies, such as pasireotide, may result in a glucose imbalance [11]. A recent review article has highlighted the underlying mechanisms for DM in acromegaly, its effect on patient morbidities and mortality, as well as the treatment modalities that are more suited for patients with acromegaly [8].

In this literature review, we aim to evaluate the impact of the different pharmacological therapies for acromegaly (first- and second-generation somatostatin receptor ligands, a GH receptor antagonist, and dopamine agonists) on glucose homeostasis and the underlying mechanisms through which they may exert these effects.

## 2. Somatostatin Receptor Ligands (SRLs)

### 2.1. First-Generation Somatostatin Receptor Ligands

First-generation somatostatin receptor ligands (fg-SRLs), octreotide and lanreotide, currently represent the first-line medical therapy for patients with acromegaly in most countries [12,13]. These are both small octapeptides designed based on the structure of naive somatostatin, showing a preferential binding affinity for somatostatin receptor subtype 2 (SST_2_) and a moderate affinity for somatostatin receptor subtype 5 (SST_5_) [14]. Pancreatic α- and β-cells variously express SSTs, with SST_2_ being mostly expressed in α-cells, and SST_5_ in β-cells [15]; therefore, fg-SRLs may impact glucose metabolism [16]. Fg-SRLs also affect incretin secretion, inhibiting glucagon-like peptide-1 (GLP-1) and gastric inhibitory polypeptide (GIP) secretion [17].

Many clinical studies and an early meta-analysis reported an overall neutral effect of fg-SRLs on glucose metabolism, with no major differences found between octreotide and lanreotide therapy [18]. In more detail, the first meta-analysis about the impact of fg-SRLs on glucose homeostasis, evaluating 619 patients with acromegaly from 31 studies (both prospective and retrospective), found a significant decrease in fasting plasma insulin [ES (effect size) −0.45 mU/L, 95% CI (confidence interval) −0.58 to −0.32 mU/L, *p* < 0.001] and an increase in plasma glucose values during the oral glucose tolerance test (OGTT) [ES +0.31 mmol/L, 95% CI +0.17 to +0.45 mmol/L, *p* < 0.001] [19]. Both disease duration and biochemical control did not affect these two parameters at meta-regression analysis [19]. Of note, the effect of fg-SRLs was neutral on fasting plasma glucose (FPG) [ES +0.04 mmol/L, 95% CI +0.07 to +0.15 mmol/L, *p* = 0.52] and glycated haemoglobin (HbA_1c_) [ES +0.11%, 95% CI +0.02% to +0.23%, *p* = 0.09] [19]. The presence of impaired glucose tolerance or DM before fg-SRL therapy did not significantly impact the hypometabolic outcome of treated patients since the analysis of 107 subjects with available data showed a worsening of glucose parameters in 25% of cases, an improvement in 29%, and unchanged values in 46% [19].

A more recent meta-analysis, evaluating 1297 patients from 47 prospective studies, used more stringent inclusion criteria (e.g., >6-month follow-up with long-acting fg-SRLs) and expanded the analysis on other parameters of glycometabolic status, such as the homeostatic model assessment (HOMA)-index, HOMA-β, triglycerides, weight, and body mass index (BMI) [20]. In line with the previous meta-analysis from Mazziotti and colleagues [19], the authors found a significant decrease in fasting plasma insulin [ES −6.67 mU/L, 95% CI −8.38 to −4.95 mU/L, *p* < 0.001], an increase in plasma glucose after the OGTT [ES +0.59 mmol/L, 95% CI +0.05 to 1.13 mmol/L, *p* = 0.032], and only a marginal effect on FPG [ES +0.06 mmol/L; 95% CI −0.06 to 0.18 mmol/L, *p* = 0.356] following fg-SRL therapy [20]. On the other hand, a slight, although statistically significant, increase in HbA_1c_ was observed [ES +0.12%, 95% CI +0.04% to +0.21%, *p* = 0.003]. This latter finding is comparable to that of Mazziotti et al. regarding the observed effect size, thus reaching statistical significance due to the greater sample size [20]. The indexes of insulin resistance and β-cell function, such as the HOMA-index [ES −1.57, 95% CI −2.42 to −0.72, *p* < 0.001] and HOMA-β [ES −47.45, 95% CI −73.15 to −21.76, *p* < 0.001], were significantly lowered by fg-SRL therapy. The observed improvement in insulin sensitivity indexes is likely related to both the direct effect on insulin secretion on β-cells and the reduction of GH levels mediated by fg-SRLs. Interestingly, fg-SRL treatment was associated with a lowering of triglyceride levels [ES −0.37 mmol/L, 95% CI −0.47 to −0.27 mmol/L, *p* < 0.001].

Another meta-analysis, by Biagetti and colleagues, included 15 studies (prospective, retrospective, and cross-sectional) with a total of 674 patients with acromegaly [21]. In the sub-analysis evaluating glucose metabolism after SRL treatment, four studies with a total of 106 patients were included; FPG concentrations and the HOMA-index were evaluated [21]. According to the previously mentioned meta-analyses, the HOMA-index significantly decreased after treatment with SRLs [ES −2.30, 95% CI −3.05 to −1.56, *p* < 0.01], while FPG levels did not significantly change [ES 0.08 mmol/L, 95% CI −0.18 to 0.34 mmol/L, *p* = 0.55] [21].

In theory, the main impact of fg-SRL therapy should be observed on postprandial glucose values, although the high heterogeneity observed in meta-analyses suggests that additional multiple factors (e.g., familial history, age, ethnicity, BMI) may dictate the balance between the positive (GH/IGF-1 reduction) and the negative (insulin reduction) effects of fg-SRLs on glucose metabolism. Overall, the effect of fg-SRLs on glucose homeostasis may be considered neutral [8].

### 2.2. Second-Generation Somatostatin Receptor Ligands

Nowadays, pasireotide (PAS) is the only second-generation SRL (sg-SRL) approved for medical treatment in acromegaly. It can be used as the second-line treatment in those patients not reaching biochemical control after first-generation SRL therapy [12,22]. PAS is a stable cyclohexapeptide showing a preferential binding affinity for SST_5_ and a remarkable affinity for SST_2_, SST_1_, and SST_3_ as well [23]. It has a 30- to 40-fold higher affinity for SST_5_ and a slightly lower affinity for SST_2_ (although within the nanomolar range) compared with both octreotide and lanreotide, as well as different biological properties compared with fg-SRLs (e.g., promoting differential SST_2_ phosphorylation and intracellular trafficking compared with octreotide) [14,24]. In addition, with its higher efficacy in reducing GH and IGF-1 levels compared with fg-SRLs, PAS has shown a detrimental effect on glucose metabolism since early studies on healthy volunteers and clinical trials in patients with acromegaly [25]. In healthy subjects, subcutaneous PAS administration (600–900 µg bid) significantly decreased OGTT-stimulated insulin secretion (AUC −61.9%, *p* < 0.001) as well as GLP-1 (−46.7%) and GIP levels (−69.8%) [26]. Glucagon secretion was less affected (−10 to −15%); therefore, a significant increase in plasma glucose was observed (+67.4%) [26]. The imbalance between insulin and glucagon secretion (likely due to the differential SST expression of pancreatic α- and β-cells) together with the dramatic decrease in GLP-1 and GIP levels represent the pathophysiological rationale for PAS-induced glucose imbalance in healthy subjects [27]. Unlike fg-SRLs, PAS results in a significant decrease in insulin levels without improvement in insulin resistance [28]. In the first phase III randomized clinical trial (C2305 study) including patients with acromegaly naïve to medical treatment, pasireotide long-acting release (PAS-LAR) induced hyperglycaemia-related adverse events (AEs) in 57.3% of patients [95% CI, 49.7–64.7%], compared with 21.7% observed in octreotide-treated subjects [95% CI, 15.9–28.4%] [25]. In the phase III PAOLA study (C2402 study), evaluating patients previously uncontrolled by fg-SRLs, hyperglycaemia-related AEs (defined as: hyperglycaemia, DM, impaired glucose tolerance, and increased blood glucose) were reported in 67% and 61% of patients treated with PAS-LAR 40 mg and 60 mg every 4 weeks, respectively [29]. Impaired glucose metabolism was reported in 30% of patients maintaining fg-SRL treatment [29]. Antidiabetic medications were initiated in 38% (PAS 40 mg), 39% (PAS 60 mg), and only 6% of patients treated with fg-SRLs [29]. Real-life studies confirmed these results, reporting glucose impairment in >60% of patients (although with high heterogeneity among study populations, e.g., glycaemic status at baseline) (see Table 1) [28,30,31,32,33,34,35,36,37,38]. A very recent meta-analysis has shown an overall negative impact of PAS-LAR on glucose parameters: the random effect model revealed a significant increase in glucose levels with a standard mean difference (SMD) of −0.8 mg/dL (95% CI −1.0 to −0.5, *p* < 0.01), as well as an increase in HbA_1c_ levels (SMD −0.5%, 95% CI −0.7 to −0.2, *p* < 0.01) and in the prevalence of type 2 DM (SMD −11.5%, 95% CI −17.5 to −5.5, *p* < 0.01) [39].

The post hoc analysis of the C2305 and C2402 studies has uncovered peculiar characteristics of PAS-induced hyperglycaemia [40]. It has a rapid onset (peak of FPG within 1–3 months), and it is largely reversible since plasma glucose and HbA_1c_ levels return to baseline 3 months after resuming therapy with fg-SRLs [28,36,40]. Identified predictors for the development of PAS-induced hyperglycaemia are: DM at baseline, higher HbA_1c_ levels, older age (>40 years), and concomitant dyslipidaemia [40]. The BMI resulted in being a predictive factor only in the post hoc analysis of the C2402 study, when comparing obese (BMI > 30 kg/m^2^) vs. normal weight patients (BMI < 25 kg/m^2^). A recent real-life study carried out on 50 patients highlighted the role of higher baseline IGF-1 values as an additional predictive factor of glucose impairment [36]. Overall, the mean HbA_1c_ increase observed in both clinical trials and real-life studies (considering the use of proper antidiabetic drugs) does not exceed +1.0% (+11 mmol/mol) [25,34]. Metformin therapy alone (together with lifestyle interventions) is a valuable first-line approach in patients needing de novo antidiabetic medications [28,40,41]. In a phase IV randomized clinical trial, 121/190 (64%) of patients treated with PAS did not need antidiabetic agents other than metformin. In detail, 88/121 patients maintained normal glucose metabolism without any specific intervention, while 33/121 patients only needed to start metformin alone [41]. Fifty-six patients were randomized to add incretin-based therapy or insulin to metformin, showing an overall statistically significant decrease in mean HbA_1c_ levels (−0.36%; 95% CI –0.74 to 0.02), with a superior efficacy observed in the incretin-based treatment arm vs. insulin [41].

### 2.3. Molecular Mechanisms of Hyperglycemia Secondary to SRL Treatment

Pancreatic islets are small endocrine organs composed of several cell types responsible for glucose control, metabolism, and eating behaviour. The main cell types involved in blood glucose control are [42]:

1. β-cells, secreting insulin after eating to suppress hepatic glucose production and to stimulate glucose uptake in several tissues, such as skeletal muscle and white adipose tissue;
2. α-cells, secreting glucagon to increase blood glucose during fasting through glucose production, mainly in the liver;
3. δ-cells, secreting the inhibitory hormone somatostatin for regulating paracrine pancreatic islet functions, such as modulation of insulin/glucagon balance. Of note, only 5% of circulating somatostatin is derived from δ-cells.

**Figure 1:**
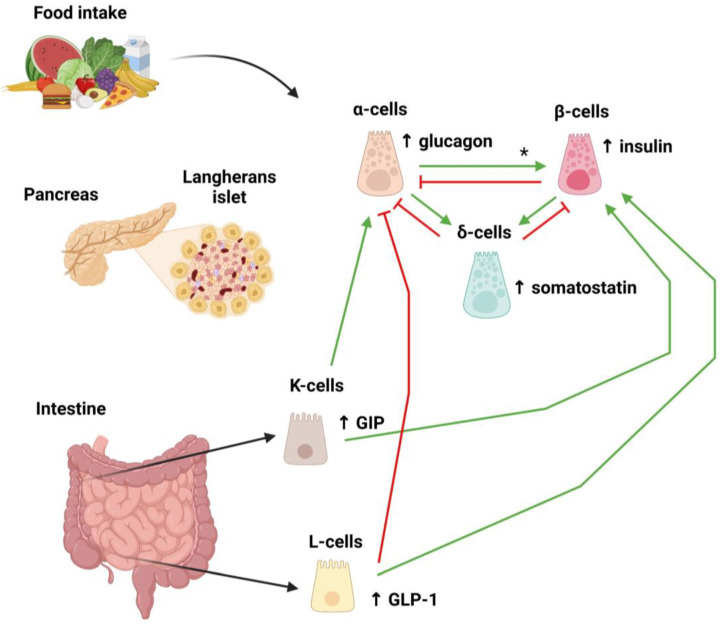
Schematic and simplified representation of the feedback loops triggered by food intake involving the endocrine pancreas and incretin secretion. Following food intake and the related increase in plasma glucose, pancreatic β-cells secrete insulin, while small intestine K- and L-cells secrete gastric inhibitory polypeptide (GIP) and glucagon-like peptide-1 (GLP-1). Both GIP and GLP-1 further stimulate insulin secretion by β-cells. Meanwhile, in order to maintain glucose homeostasis, a complex negative feedback loop involving both glucagon and somatostatin (the latter secreted by δ-cells) is activated. Red lines: inhibitory signal; green arrows: stimulatory signal. * Stimulatory signal from α- to β-cells promoted by proglucagon-like peptides.

These neighbouring cells are surrounded by a capillary network that allows hormones to control glucose homeostasis acting throughout a finely tuned paracrine signalling pathway (Figure 1) [42]. Pancreatic islet cells are mutually regulated to control glucose homeostasis. On the one hand, β-cells through the release of insulin and γ-aminobutyric acid (GABA) inhibit glucagon secretion by α-cells while, on the other hand, they promote somatostatin exocytosis throughout the stimulatory mediators urocortin-3 and GABA, which act on specific receptors expressed on δ-cells, and by electrical coupling via gap junctions [43]. Likewise, α-cells release glucagon, which stimulates somatostatin secretion by activating adenylyl cyclase and proglucagon-like peptides while increasing glucose-stimulated insulin secretion as a part of a negative feedback loop [42]. On the other hand, somatostatin released by δ-cells inhibits both insulin and glucagon secretion [42]. Other cells outside the pancreatic islets, such as the enteroendocrine L- and K-cells, play an important role in glucose homeostasis. The former are located most densely in the colon and rectum and secrete GLP-1, while the latter are mostly represented in the duodenum and produce GIP [44,45].

The release of insulin and glucagon share common mechanisms, including the stimulation of voltage-gated calcium channels that raise intracellular calcium concentrations and trigger hormone exocytosis [46]. The somatostatin inhibitory effect is mediated by activating inhibitory G proteins (Gi) coupled to somatostatin receptors, which are widely expressed on α- and β-cells, thus reducing the activity of adenylyl cyclase, lowering cyclic adenosine monophosphate (cAMP) production and protein kinase A activity, and stimulating phospholipase C and subsequent intracellular calcium mobilisation [47]. In rodents, SST_2_ is predominantly expressed in α-cells, while SST_5_ is the most represented SST subtype in the β-cells; selective targeting of these receptors results in the inhibition of glucagon and insulin secretion, respectively [48]. In line with these findings, SST_5_ knockout mice display increased basal insulin secretion [49].

**Figure 2:**
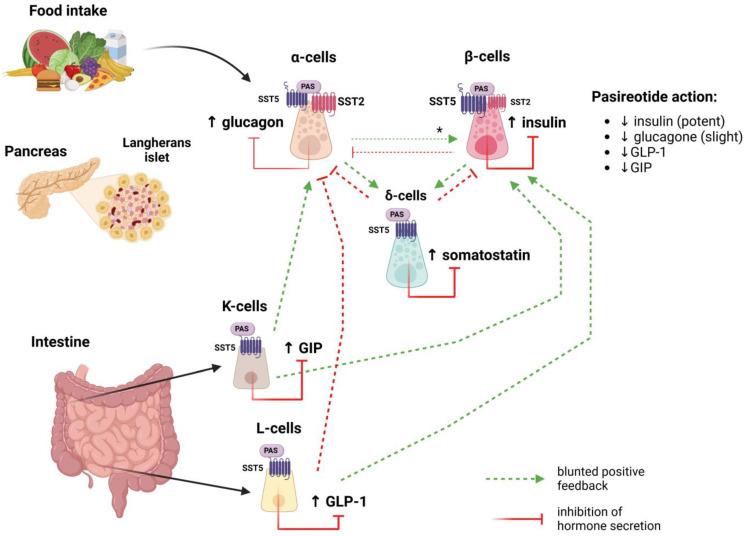
Schematic and simplified representation of the mechanisms underlying pasireotide effects on glucose metabolism. Pasireotide treatment can significantly impact glucose metabolism, acting both on pancreatic islet cells and intestinal K- and L-cells. Throughout its potent binding affinity for SST_5_, the compound results in a significant decrease in insulin, GLP-1, and GIP levels, which are not fully counterbalanced by a concomitant strong decrease in glucagon levels. * Stimulatory signal from α- to β-cells promoted by proglucagon-like peptides. Abbreviations: SST_2_: somatostatin receptor subtype 2; SST_5_: somatostatin receptor subtype 5; PAS: pasireotide.

In human pancreatic islets, locally produced somatostatin inhibits hormone secretion from both α- and β-cells mainly acting through SST_2_ targeting [50]. Previous studies showed that selective SST_2_ agonists inhibit both insulin and glucagon secretion with similar potency (EC50: 0.08 nM and 0.05 nM, respectively), while selective SST_5_ agonists inhibit insulin secretion with higher potency (EC50: 5.3 nM) than glucagon (EC50: 28 nM) [51]. The inhibitory effect of somatostatin on insulin and glucagon secretion is crucial to maintaining the β-cell threshold for glucose-induced insulin release [52]. Interestingly, SST_5_ is also expressed in the enteroendocrine L- and K-cells, where somatostatin suppresses cAMP-induced GIP and GLP-1 secretion acting through SST_5_ binding [53] (Figure 2). GLP-1 plays a pivotal role in glucose homeostasis; it is mainly produced by enteroendocrine L-cells, although pancreatic α-cells also contribute to its secretion, suggesting paracrine actions on the neighbouring β-cells [54]. However, the impact of pancreatic GLP-1 on glucose homeostasis is still debated [55,56]. The blood glucose-lowering action of GLP-1 is a glucose-dependent function: GLP-1 reduces blood glucose levels only when hyperglycaemia is present, such as after a meal, while as the postprandial blood glucose levels fall in response to GLP-1, the action of GLP-1 is self-terminating without leading to hypoglycaemia [57]. GLP-1 also acts locally within the intestinal wall to control gastric motility, slowing gastric emptying, thus enhancing satiety and reducing food intake. In the pancreatic islets, GLP-1 induces insulin release and suppresses glucagon secretion [58]. Thus, in addition to the higher inhibitory effect exerted on insulin secretion compared with glucagon, SST_5_ agonists may indirectly regulate plasma glucose levels by affecting GLP-1 release, and SST_5_-targeting drugs may result in hyperglycaemia.

The tissue distribution of SST_5_ may explain the relationship between somatostatin, insulin, and GLP-1. Indeed, somatostatin is also produced by the D-cells in the ileum, which are close to the L-cells [59]. This allows a finely tuned mechanism of paracrine regulation between the secretion of somatostatin and GLP-1 within the gastrointestinal tract: GLP-1 stimulates somatostatin secretion that, in turn, inhibits GLP-1 release [60,61]. This negative feedback loop occurs through the activation of SST_5_ on L-cells, adding to the importance of this SST subtype in regulating glucose homeostasis. Of note, it has been reported that SST_5_ antagonists can increase GLP-1 secretion, and SST_5_ knockout mice show higher GLP-1 plasma levels compared with wild-type controls [62,63]. Furthermore, treatment with SST_5_ antagonists lowers glucose levels by stimulating glucose-induced GLP-1 secretion and may indirectly promote insulin secretion [62,64]. Considering all this evidence, it is easier to understand PAS-induced glucose imbalance due to the high affinity of this compound for SST_5_. On the other hand, the higher binding affinity of octreotide and lanreotide for SST_2_ compared with SST_5_ leads to the inhibition of glucagon secretion with a more balanced impact on insulin release, thus resulting in an (overall) neutral impact on glucose homeostasis. The impaired secretion of GLP-1 following PAS treatment may affect glucose homeostasis by directly reducing insulin secretion, as well as suppressing the inhibitory effect of GLP-1 on food intake [65]. In this light, antidiabetic drugs with incretin-based mechanisms of action, such as GLP-1 receptor agonists (GLP-1 RAs) and dipeptidyl peptidase-4 inhibitors (DPP-4i), have been demonstrated to be effective in treating PAS-induced hyperglycaemia [66].

## 3. Growth Hormone Receptor Antagonists

Pegvisomant (PEG) is a genetically engineered, recombinant GH receptor antagonist [67] that can be used as the second-line medical therapy when the maximal tolerated dose of first-generation SRLs is not successful in normalizing IGF-1 levels [12]. PEG has demonstrated a more favourable effect on glucose metabolism than fg-SRLs in patients with acromegaly, lowering FPG and HbA_1c_ levels and improving glucose tolerance [68]. It also improves peripheral and hepatic insulin sensitivity and reduces basal lipolysis and the endogenous glucose production rate, which reflects hepatic glucose output [69,70]. A recent meta-analysis evaluated the metabolic changes that were induced by PEG when it was used either as monotherapy (13 studies) or combined with fg-SRLs (5 studies) in a total of 550 patients with acromegaly (Table 2). PEG has a positive impact on glucose metabolism, inducing a significant decrease in FPG [ES −0.80 mmol/L, 95% CI, −1.06 to −0.55, *p* < 0.001] and, accordingly, a marked decrease in HbA_1c_ values [ES −0.43%, 95% CI −0.56 to −0.31, *p* < 0.001]. It also induces a decrease in fasting plasma insulin [ES −5.31 mU/L, 95% CI −10.23 to −0.39, *p* = 0.034] and a significant reduction of the HOMA-index [ES −0.61, 95% CI −1.17 to −0.04, *p* = 0.034] [71]. The addition of PEG to fg-SRLs seems to mitigate the effects of fg-SRLs on glucose metabolism, producing an overall neutral effect, except for the observed decrease in fasting plasma insulin [ES −3.63 mU/L, 95% CI −4.11 to −3.14, *p* < 0.001] [71]. Of note, the effects of PEG on glucose metabolism parameters did not seem to be correlated with the changes in IGF-1 concentration [71]. Different potential mechanisms have been hypothesized to explain PEG action, which may involve the blockade of the GH receptor on peripheral tissues, especially in the liver, the muscles, and the adipose tissue, directly counteracting the detrimental effect of GH on insulin signalling, lipolysis, and gluconeogenesis. Furthermore, the beneficial effect due to fg-SRL discontinuation and/or dose reduction, after PEG start, may play an additional role in this setting. The ACROSTUDY, a multicentre, non-interventional study including 2221 patients with acromegaly treated with PEG for a median of 9.3 years (range, 0–20.8 years), followed up for a median of 7.4 years (range, 0–13.9 years), has evaluated the impact of PEG on glucose metabolism [72]. An earlier, 4-year, longitudinal interim analysis of ACROSTUDY (evaluating 1762 patients) explored the impact of PEG in patients with diabetes, where the mean blood glucose decreased by 20.2 mg/dL from baseline to year 4, while the mean HbA_1c_ remained unchanged [73]. At the 9-year analysis, out of 996 patients (44.8%) with a glucose value < 200 mg/dL at PEG start, only 29 (2.9%) had a reported glucose value > 200 mg/dL during treatment, while of the 540 patients with a baseline HbA_1c_ < 6.5%, only 80 (14.8%) had at least one HbA_1c_ value > 6.5% reported during PEG therapy [72].

To date, whether PEG can contrast the (potential) detrimental effect of fg-SRLs on glucose metabolism, when used in combination, has not yet been fully clarified. A recent meta-analysis (9 studies) demonstrated that combination therapy with PEG + fg-SRLs did not significantly affect FPG [ES 0.011 mmol/L, 95% CI −0.374 to 0.397 mmol/L, *p* = 0.954] or HbA_1c_ [ES −0.074%, 95% CI −0.166 to 0.315%, *p* = 0.544], while a significant decrease in fasting plasma insulin was reported [ES −21.487 pmol/L, 95% CI −35.713 to −7.260 pmol/L, *p* = 0.003] [74].

## 4. Dopamine Agonists

Bromocriptine, cabergoline, and quinagolide are commercially available dopamine agonists used in the treatment of endocrine conditions such as hyperprolactinemia and acromegaly. Bromocriptine and cabergoline (both ergot derivatives [80,81]), as well as quinagolide (a non-ergot derivative), act on binding with a preferential affinity to the dopamine receptor subtype 2 (D2DR), which is widely expressed, among others, on lactotroph and somatotroph cells [82]. Nowadays, the use of dopamine agonists in acromegaly has been overshadowed by more effective drugs, such as fg-SRLs, PEG, and PAS [9,12]. Cabergoline is still considered as off-label treatment in patients with mild disease activity (IGF-1 levels < 2.5 times the upper limit of normal) after surgery and as add-on therapy in selected cases, such as in patients who do not achieve biochemical control with maximal doses of fg-SRLs or PEG [9]. Of note, both bromocriptine and cabergoline have shown a favourable effect on glucose metabolism in patients with acromegaly (Table 2) [27,75,76]. Particularly, earlier studies demonstrated a beneficial effect of bromocriptine in improving glucose homeostasis, describing the reversal of impaired glucose tolerance and DM during treatment and reduced fasting and 2h-OGTT glucose levels, as well as decreased fasting and glucose-stimulated insulin levels [83]. As concerns cabergoline, a multicentre prospective clinical study showed that cabergoline therapy normalized glucose homeostasis in 4 out of 7 (57%) patients with impaired glucose tolerance or DM at baseline and significantly reduced 2h-OGTT glucose levels in the entire cohort (24 patients) [79]. Another study showed that adding cabergoline to PEG therapy significantly decreased glucose rise after meals [78]. Overall, the improved glucose tolerance observed during dopamine agonist treatment is not always correlated with reduced GH and/or IGF-1 levels [27]. Indeed, it is well known that dopamine agonists directly improve glucose metabolism in different clinical conditions beyond acromegaly. Several studies have demonstrated that bromocriptine improves glucose homeostasis in patients with DM and/or obesity [84]. Multiple mechanisms have been hypothesized to explain this action, which may involve the activity on food intake control, a reduced noradrenaline output, and the regulation of circadian rhythm at the central nervous system, as well as the decrease in prolactin secretion at the pituitary level through decreasing lipolysis, insulin resistance, and gluconeogenesis [77,85]. A direct effect on insulin secretion in the periphery can also be hypothesized since D2DR is expressed in both pancreatic β-cells and adipocytes. Based on the previously mentioned findings, a short-acting (circadian-timed) bromocriptine formulation has been approved by the Food and Drug Administration for the treatment of DM type 2 [86].

## 5. Future Therapies for Acromegaly

### 5.1. Paltusotine

Paltusotine (formerly CRN00808) is an oral selective nonpeptide small SST_2_ agonist with transcellular absorption [87]. A number of phase II and III studies have been completed, while others are still ongoing. ACROBAT Edge (NCT03789656) is an open-label exploratory single-arm phase II study aimed at evaluating the safety, efficacy, and pharmacokinetics of switching patients with acromegaly from injectable SRLs to paltusotine. Forty-seven patients were treated with paltusotine monotherapy for a 13-week treatment period (starting dose 10 mg/day with subsequent increases of 10 mg up to a maximum of 40 mg/day). During the study, there was no evidence for a deterioration of glucose metabolism as measured by serum glucose or HbA_1c_ levels [88]. ACROBAT Evolve (NCT03792555) is a double-blind, placebo-controlled, randomized withdrawal phase II study that evaluated the safety, efficacy, and pharmacokinetics of paltusotine in 13 subjects with acromegaly who were responders to injectable fg-SRLs. Only one subject (7.69%) had an increase in blood glucose during the titration period of paltusotine. ACROBAT Advance (NCT04261712), a long-term extension phase II study of ACROBAT Edge and ACROBAT Evolve study, evaluating the incidence of treatment-emergent adverse events as primary endpoint, is still ongoing. Results of PATHFNDR-1 (NCT04837040), a randomized, placebo-controlled phase III study designed to evaluate the safety and efficacy of paltusotine in subjects with acromegaly previously treated with fg-SRLs with controlled disease, have been recently published. Fifty-eight patients were randomized to paltusotine or placebo for 36 weeks: one patient in the paltusotine group and two patients in the placebo group had hyperglycaemia as recorded adverse events [89]. PATHFNDR-2 (NCT05192382), a randomized, placebo-controlled phase III study designed to evaluate the safety and efficacy of paltusotine in subjects with non-pharmacologically treated acromegaly, is still ongoing, and results have not yet been published.

### 5.2. Somatoprim

Somatoprim, also known as veldereotide (or DG3173 or COR-005), is a subcutaneous SRL with preferential binding affinity for SST_2_, SST_4_, and SST_5_ [90] that, in vitro, demonstrated a similar inhibitory effect on GH secretion compared with octreotide [91]. No impairment of glucose metabolism was detected in two phase II studies (NCT02235987 and NCT02217800), whose results are available on ClinicalTrials.gov.

### 5.3. ONO-5788 and ONO-ST-468

ONO-5788 and ONO-ST-468 are two new SST_2_ selective agonists [90]. ONO-5788 has an active metabolite (ONO-ST1-641) that showed a greater agonist effect on SST_2_ than octreotide and pasireotide in vitro and a potent inhibition of GH secretion in primary cultures of human somatotropiomas, as well as in mouse models [92,93]. The molecule is administered orally without significant effects on insulin secretion [90]. Two phase I clinical trials in healthy adult volunteers have been completed in order to evaluate the safety, tolerability, and pharmacokinetics of ONO-5788 (NCT03849872 and NCT03571594); however, no results are available yet. To date, ONO-ST-468 has no known effect on glucose metabolism, as no clinical trials have been conducted in human subjects so far.

## 6. Treatment of Acromegaly Treatment-Related Diabetes

According to the American Diabetes Association, DM secondary to acromegaly is classified as a specific type of diabetes [94], yet no specific recommendations or guidelines for its treatment exist to date. A recent review has discussed the treatment modalities for DM more suited for patients with acromegaly [8]. The same treatment strategies for patients with “classical” type 2 DM can be used in patients with acromegaly-related and acromegaly treatment-related DM. As noted, while the GH receptor antagonist and the dopamine agonists can positively impact glucose metabolism, the fg-SRLs show a neutral effect. Meanwhile, the sg-SRL pasireotide can negatively affect glucose homeostasis therefore, initiating PAS may necessitate an adjustment or the beginning of antidiabetic therapy. Various antidiabetic drugs in patients experiencing PAS-induced hyperglycaemia have proven effective in preventing glycaemic alterations and in reducing HbA_1c_ variability during treatment [41]. If not contraindicated, metformin should be regarded as the first-line medical therapy [8,41]. In case of ineffectiveness or intolerance to metformin, drugs with incretin-based mechanisms of action, such as GLP-1 RAs and DPP-4 inhibitors, should be considered. Particularly in patients with pasireotide-induced hyperglycaemia, these drugs demonstrated greater efficacy in reducing HbA_1c_ than insulin [41]. These medications function by stimulating insulin production in a glucose-dependent manner, while inhibiting glucagon production, slowing gastric motility, and promoting appetite suppression, which can contribute to weight loss [95]. Additionally, GLP-1 RAs have been shown to decrease major cardiovascular events in patients with type 2 DM [96,97].

## 7. Conclusions

Glucose intolerance and diabetes mellitus are common metabolic complications in acromegaly, with a huge impact on patients’ morbidity and mortality. Acromegaly treatment, through lowering plasma GH and IGF-1 concentrations, should lead to an improvement in glucose metabolism. However, medical therapies for acromegaly have different effects on glucose homeostasis. First-generation SRLs (octreotide and lanreotide) show an overall neutral effect on the main parameters of glucose metabolism, whereas the second-generation SRL pasireotide can have a detrimental effect on glucose homeostasis, although fully reversible at discontinuation of treatment. Both the GH receptor antagonist pegvisomant and the clinically available dopamine agonists (cabergoline and bromocriptine) seem to have a favourable effect on glucose metabolism; therefore, these drugs could be considered in treating patients with acromegaly and associated glucose imbalance/DM. Further studies are needed to more deeply investigate the effects of current therapies for acromegaly on glucose homeostasis, particularly studies with a larger sample size and a possibly longer follow-up period, as well as studies on new drugs (e.g., paltusotine, somatoprim, ONO-5788, and ONO-ST-468).

## Figures and Tables

**Table 1:** The impact of pasireotide therapy on glucose metabolism parameters in patients with acromegaly *.

	Study Design	Number of Patients	∆ FPG (mg/dL)	∆ Glucose Load (mmol/L)	∆ HbA_1c_ (%)	∆ FPI (mU/L)	∆ HOMA-IR
Shimon et al., 2018 [30]	Retrospective study	35	↑ 29 mg/dL	-	↑ 0.6	-	-
Lasolle H et al. 2019 ^a^ [31]	Prospective study	15	↑ 16 mg/dL	-	↑ 0.5	-	-
Akirov et al. 2021 ^b^ [32]	Retrospective study	19	-	-	↑ 0.5	-	-
Witek et al., 2021 [33]	Retrospective study	39	-	-	↑ 0.40 (−0.20; 2.30)	-	-
Wolf et al., 2022 [28]	Retrospective study	33	-	↑ 250 (AUC mmol/L × min) ^c^	↑ 1.0	-	↓ −0.32 ^c^
Stelmachowska-Banaś et al., 2022 [34]	Prospective study	28 (26 enrolled for the final analysis)	↑ 14.99 mg/dL (8.55; 21.42)	-	↑ 0.53 (0.38; 0.68)	-	-
Corica et al., 2023 ^a^ [35]	Retrospective study	21	↑ 31 mg/dL	-	↑ 0.3	-	-
Gadelha et al., 2023 [36]	Retrospective study	54	↑ 16 mg/dL	-	↑ 1.3	-	-
Pirchio et al., 2024 [37]	Retrospective study	28	↑ 17 mg/dL ^d^	-	↑ 0.1 ^d^	↓ −3.6 ^d^	↓ −0.6 ^d^
Urbani et al., 2024 [38]	Retrospective study	50	↑ 22 mg/dL ^e^	-	↑ 2.8 ^e^	-	-

Legend: FPG, fasting plasma glucose; HbA_1c_, glycosylated haemoglobin; FPI, fasting plasma insulin; HOMA-IR, homeostatic model assessment of insulin resistance; -, no available data; ∆, delta; ↑, increase; ↓ decrease. * The table reports findings from available clinical studies on the impact of pasireotide on glucose metabolism. Studies assessing the main changes on FPG, glucose load, HbA_1c_, fasting plasma insulin, and HOMA-IR have been reported. ^a^ Patients switched to pasireotide from combined medical therapies or unconventional dosages of fg-SRLs. ^b^ Long-term evaluation on a subset of patients already reported in the study by Shimon et al., 2018 [30]. ^c^ Data available for 14 out of 33 patients included in the study. ^d^ Data at last evaluation (36 months) available for 18 out of 26 patients. ^e^ Data available at last visit.

**Table 2:** The impact of pegvisomant and dopamine agonists on glucose metabolism in patients with acromegaly (alone or in combination) *.

	Study Design	Number of Patients	Treatment	∆ FPG (mmol/L or mg/dL)	∆ Glucose Load (mmol/L)	∆ HbA_1c_ (%)	∆ FPI (mU/L)	∆ HOMA-IR
**Pegvisomant**								
Feola et al., 2019 [71]	Meta-analysis (16 studies)	550	PEG monotherapy	↓ −0.80 (−1.6; −0.55) mmol/L	↓ −2.75 (−5.91; 0.41)	↓ −0.43 (−0.56; −0.31)	↓ −5.31 (−10.23; −0.39)	↓ −0.61 (−1.17; −0.04)
			PEG + fg-SRL	↓ −0.09 (−0.58; −0.40) mmol/L	-	↓ −0.12 (−0.24; 0.00)	↓ −3.63 (−4.11; −3.14)	↓ −0.98 (−2.33; 0.37)
Ma et al., 2020 [74]	Meta-analysis (9 studies)	318	PEG + fg-SRL	↑ 0.011 (−0.374; 0.397) mmol/L	-	↓ −0.074 (−0.166; 0.315)	↓ −21.487 (−35.71; −7.26)	-
**Dopamine agonists**								
Wass et al., 1980 [75]	Cross-sectional	69	Bromocriptine	-	↓	-	-	-
Feek et al., 1981 [76]	Prospective study	12	Bromocriptine	↓	↓	-	↓	-
Rau et al., 1993 [77]	Prospective study	12	Bromocriptine	↓	↓	-	-	-
Roemmler et al., 2010 [78]	Cross-sectional	9	Cabergoline + PEG vs. PEG alone	-	↓ (both peak glucose and AUC glucose) ^a^	-	-	-
Higham et al., 2012 [79]	Prospective study	24	Cabergoline ^b^	↓ −0.4 mmol/L	↓ −2.2	↓ −0.20	-	-

Legend: FPG, fasting plasma glucose; HbA_1c_, glycosylated haemoglobin; FPI, fasting plasma insulin; HOMA-IR, homeostatic model assessment of insulin resistance; PEG, pegvisomant; fg-SRL, first-generation somatostatin receptor ligand; -, no available data; ∆, delta; ↑, increase; ↓ decrease. * The table reports findings from available meta-analyses and clinical studies on the impact of the GH receptor antagonist, pegvisomant, and the dopamine agonists, bromocriptine and cabergoline, on glucose metabolism. No meta-analysis on the effect of dopamine agonists has been performed so far. Therefore, findings from studies assessing the main changes on FPG, glucose load, HbA_1c_, fasting plasma insulin, and HOMA-IR have been reported. ^a^ Single administration of cabergoline; evaluation after mixed meal. ^b^ Cabergoline up-titration up to 18 weeks (maximum dose of 0.5 mg once daily); then, PEG was added.

## Data Availability

Data are contained within the article.

## Abbreviations

BMI: body mass index; CI: confidence interval; DM: diabetes mellitus; DPP-4i: dipeptidyl peptidase-4 inhibitor; D2DR: dopamine receptor subtype 2; ES: effect size; fg-SRLs: first-generation somatostatin receptor ligands; FPG: fasting plasma glucose; GH: growth hormone; GHRH: growth hormone-releasing hormone; GIP: gastric inhibitory polypeptide; GLP-1: glucagon-like peptide-1; GLP-1 RA: glucagon-like peptide-1 receptor agonists; HbA_1c_: glycated haemoglobin; HOMA: homeostatic model assessment; IGF-1: insulin-like growth factor-1; OGTT: oral glucose tolerance test; PAS: pasireotide; PEG: pegvisomant; sg-SRL: second-generation somatostatin receptor ligand; SST_2_: somatostatin receptor subtype 2; SST_4_: somatostatin receptor subtype 4; SST_5_: somatostatin receptor subtype 5.
